# Severe exercise-induced anaphylaxis in a hot and humid area successfully treated with omalizumab: a case report

**DOI:** 10.3389/falgy.2023.1228495

**Published:** 2023-07-27

**Authors:** Hassan Mobayed, Maryam Ali Al-Nesf, Karla Robles-Velasco, Ivan Cherrez-Ojeda, Luis Felipe Ensina, Marcus Maurer

**Affiliations:** ^1^ Allergy and Immunology Division, Department of Medicine, Hamad Medical Corporation, Doha, Qatar; ^2^ Respiralab Research Group, Guayaquil, Ecuador; ^3^ Universidad Espíritu Santo, Samborondón, Ecuador; ^4^ Division of Allergy, Immunology, Rheumatology, Department of Pediatrics, Federal University of São Paulo, São Paulo, Brazil; ^5^ GA^2^LEN Urticaria Center of Reference and Excellence (UACRE), Institute of Allergology, Charité-Universitätsmedizin Berlin, Corporate Member of Freie Universität Berlin and Humboldt-Universität zu Berlin, Berlin, Germany; ^6^ Fraunhofer Institute for Translational Medicine and Pharmacology ITMP, Allergology and Immunology, Berlin, Germany

**Keywords:** anaphylaxis, exercise-induced anaphylaxis, omalizumab, diagnosis and management, co-factors

## Abstract

Exercise-induced anaphylaxis (EIA) is a rare disorder in which anaphylaxis occurs exclusively after physical activity. Here, we report a case of severe EIA where anaphylaxis was initially only induced by strenuous exercise. Suddenly the anaphylaxis got out of control to the degree that usual daily activities triggered it. Exposure to a hot and humid environment appeared to be a cofactor for the development of severe symptoms resistant to usual preventive measures. Treatment with omalizumab (anti-IgE) was initiated and resulted in marked improvement. We discuss unique aspects of this case in comparison to published information on the clinical features, triggering cofactors, diagnosis, and treatment of EIA.

## Introduction

Anaphylaxis is the most severe clinical presentation of acute systemic hypersensitivity reactions. It is defined as a potentially life-threatening generalized or systemic hypersensitivity reaction involving several organs. Anaphylaxis most commonly comes with simultaneous involvement of the skin, mucosal tissue, or both (e.g., generalized wheals, pruritus or flushing, swollen lips-tongue-uvula) plus respiratory manifestations, circulatory compromise, and/or severe gastrointestinal symptoms. The definition also includes acute onset of hypotension or bronchospasm or laryngeal involvement after exposure to a known or highly probable allergen (1). Food, drugs, and insect venoms are common elicitors of anaphylaxis. In contrast, exercise-induced anaphylaxis (EIA) is rare (2, 3).

EIA occurs exclusively after physical exertion, without relation to food intake (4). In food-dependent exercise-induced anaphylaxis (FDEIA), a similar and related disorder, signs and symptoms of anaphylaxis only develop when physical activity takes place within a few hours of eating and usually only if certain types of food are ingested (5). FDEIA is more common than EIA, and both females and males can be affected, but it seems more occurring in females (6). EIA and FDEIA have been reported in patients of all ages (7), but most often affect adolescents and young adults (5). EIA and FDEIA can occur in patients of all ethnicities, but most reports on EIA and FDEIA are from Europe (3), North America (5, 6), or Japan (8). Reports from countries with a hot and humid climate are rare, although cases of FDEIA, but not EIA, were reported in the Middle East (9).

The management of EIA and FDEAI includes avoidance of triggers and cofactors, the prophylactic use of non-sedating antihistamines, and adrenaline injection when anaphylaxis occurs. Patients who continue to experience anaphylaxis episodes despite these measures can show a severe quality of life impairment, and the use of off-label omalizumab treatment can be successful in these cases (10–12).

Here, we report the first case of EIA from the Middle East, and we discuss how the outcomes of our diagnostic approach and therapeutic measures complement the little published information available on this condition.

## Case description

A 38-year-old female patient presented to the emergency room (ER) on 26th August 2019 with generalized hives (wheals), pruritus, and palpitations. The patient was light-headed and dizzy, and unable to stand. These signs and symptoms had started after a 30-minute fast walk. There was no breathing difficulty, cough, wheezing, chest tightness, or gastrointestinal complaints, but her systolic blood pressure (BP) was 50 mmHg. She was, therefore, treated for anaphylaxis with 0.3 mg of intramuscular epinephrine, 50 mg of intramuscular diphenhydramine, 200 mg intravenous hydrocortisone, and 2 liters of normal saline. She responded well to this treatment and was discharged with 0.3 mg IM epinephrine autoinjector for use as needed during new attacks, advised to use fexofenadine 180 mg and oral prednisone 50 mg daily for 5 days, and referred to our allergy outpatient clinic for further evaluation.

When we assessed the patient, she reported 2 similar episodes in the last 6 months, one was on April 2019 after 20 min of running, and the other was on July 2019 after climbing stairs to the 6th floor. None of her episodes were preceded by eating food, taking NSAIDs, or alcohol intake, and there was no relation to her menstrual period. The patient never had asthma, rhinitis, or food allergy. Her family history of EIA was negative. Laboratory investigations showed normal results, including complete blood count and differential, renal and liver functions, thyroid function, c-reactive protein, spirometry, and chest x-ray. Baseline serum tryptase was 4.4 ng/ml, total IgE was 26 IU/ml, and blood tests for specific IgE to common foods, including milk, wheat, peanut, tree nuts, fish, shellfish, egg, soybeans, sesame, and legumes and common inhaled allergens as well as latex were all negative. Based on that, the patient was diagnosed with EIA and advised to avoid strenuous exercise, to continue light exercise as tolerated, to carry an epinephrine autoinjector at all times, and to exercise with a partner trained in the use of the epinephrine autoinjector. In addition, she was instructed to avoid food, NSAIDs, and alcohol intake for at least 4 h prior to exercise. She was advised to immediately stop the exercise at the first signs of flushing, itching, and hives, self-administer epinephrine, and seek immediate medical assistance.

## Diagnostic assessment

**Figure 1:**
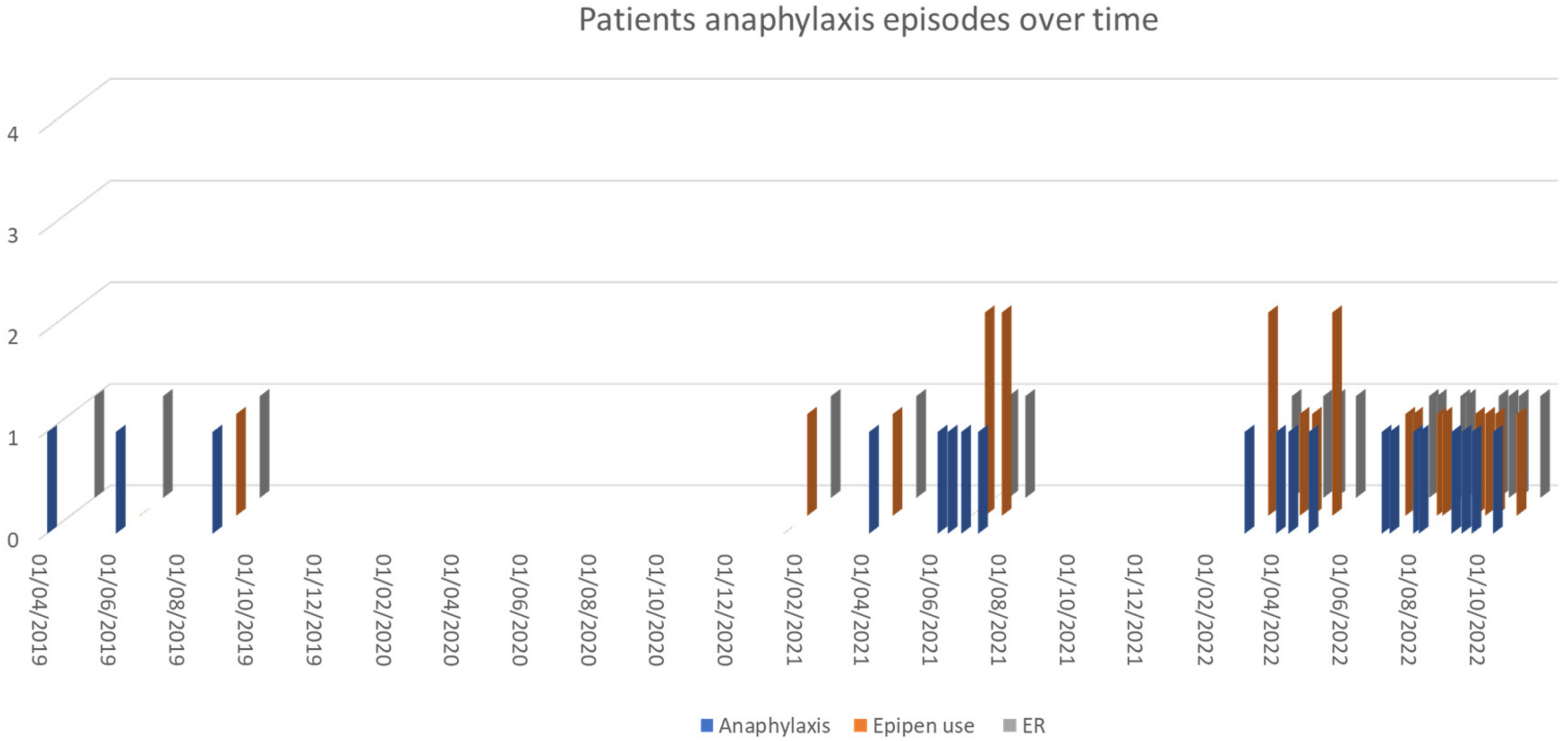
Patient's anaphylaxis episodes since diagnosis in 2019 documented ER medical records.

Our patient followed these instructions and did well for 3 years with occasional reactions, i.e., two attacks on 15th January and 1st April 2021 requiring the use of EpiPen and ER visits, few milder reactions in the first few weeks of June 2021 required antihistamine and no EpiPen or emergency visits and additional episodes on 22nd June 2021, and 7th July 2021 required the use of EpiPen and ER visit (Figure 1). The patient lives in Qatar, which has a hot and humid climate, especially in the summer months (July to September) and the start of warmer weather begins in March (13). During the summer of 2022, her trigger threshold for anaphylaxis changed markedly, and she began to have frequent episodes of anaphylaxis (10–12 per month) after light exercise and sometimes even after usual daily activities, such as walking for a few minutes from the parking lot to her office and almost all of them required the use of epinephrine autoinjectors; however not all of them attended to ER. Once, she developed severe anaphylaxis while walking at the airport to the departure gate, collapsed, and was treated with 2 injections of adrenaline in early March 2022. All anaphylaxis episodes documented in ER records are described in Figure 1.

Because of the high frequency of attacks and the severe impairment in her quality of life, we reassessed the patient by taking a detailed medical history and with new blood work, but we could not identify any new relevant cofactors or comorbidities. During one episode of anaphylaxis, she experienced chest pain, but the cardiology assessment did not reveal arrhythmia, valvular, myocardial, or coronary artery diseases, and there was no postural orthostatic tachycardia syndrome. A treadmill-based maximal exercise test using the Bruce protocol was carried out, and there was no evidence of ischemia, and she did not develop symptoms of anaphylaxis during the exercise test.

An attempt to increase Levocetirizine to 2 tablets (10 mg) per day was initiated on 20th April 2022, and montelukast 10 mg daily and the recommendation to slow down during walking were not successful in improving the frequency of the attacks. We then initiated off-label treatment with omalizumab, an anti-IgE antibody, at 300 mg subcutaneously every 4 weeks on 4th October 2022. Seven days after the first injection, our patient reported that she stopped experiencing anaphylaxis attacks and had almost returned to her normal life. Follow-up at 5 months after initiating omalizumab injections showed no more episodes of anaphylaxis despite regular physical exercise.

## Discussion

Cases of EIA have been reported worldwide, and our case shares similarities with previous ones, but also includes novel features. The latter include the sudden increase of disease activity and a drop in trigger threshold after years of stable disease, a possible seasonal influence on disease activity, and the extremely fast complete response to omalizumab treatment.

Episodes of symptoms in EIA are unpredictable in most patients, so a given level of physical exertion may trigger symptoms on one occasion but not on other similar occasions. Many patients exercise routinely but develop attacks only occasionally. Our patient was used to walking in the shopping mall 5–6 times per month, but developed anaphylaxis only once, prior to the increase in disease activity last summer. Symptoms may be triggered by vigorous exercise and sports such as jogging, football, aerobics, and dancing (5). However, episodes can be triggered in some patients by brisk walking or yard work, or just crossing a road (7). During the first few years after the onset of EIA, our patient used to have 1–2 episodes per year, some of which were minor in severity, induced by running or fast walking. During the summer of 2022, she had frequent and severe episodes of anaphylaxis triggered by walking at a normal pace for a few minutes.

Early/prodromal symptoms of EIA typically include a sensation of diffuse warmth and/or flushing, generalized pruritus, wheals (usually 10–15 mm in diameter or larger), and sudden fatigue. Cessation of exertion usually results in an improvement in symptoms. In contrast, the continuation of exercise may lead to more severe symptoms, including angioedema of the face, extremities, and/or laryngeal edema, gastrointestinal symptoms (nausea, abdominal cramping, and diarrhea), hypotension, and/or collapse (14). Shadick et al., in a previous study on EIA, reported an average rate of 8.3 attacks per year (15). Our patient had a lower attack rate during the first years of having EIA, and she then began to have very frequent episodes (10–12 per month) of anaphylaxis, with symptoms progressing to hypotension, although she stopped walking or moving at the earliest signs of a reaction.

Some patients with EIA exhibit relevant cofactors for developing anaphylaxis, such as the intake of nonsteroidal anti-inflammatory drugs (NSAIDs) or the consumption of alcoholic beverages (14). The occurrence of episodes of EIA has also been linked to the premenstrual or ovulatory phases of the menstrual cycle, seasonal pollen exposure in pollen-sensitized patients, and extremes of temperatures (15). Wade and coworkers reported an epidemiological association of EIA with a warm environment (64% of cases), high humidity (32%), and cold environment (23%) (16). Shadick et al. reported that more than one-third of EIA patients avoided exercising during extremely warm or cold temperatures or periods of high humidity to reduce the occurrence of reactions (15). Our patient, during the first years of her disease, did not have relevant cofactors of her EIA and her disease was well controlled by avoiding strenuous physical exercise. Then, in the summer season (July-September) of 2022, high temperatures (45°C–49°C) and humidity (85% to >90%) (13) became a very relevant cofactor for our patients, with a marked increase in attack frequency and anaphylactic episodes triggered by mild exercise and even usual daily activities. In our patient, why soaring temperature and high humidity evolved as EIA cofactors over time is not fully understood. It was suggested that the cofactor's role in eliciting anaphylaxis is increasingly accepted. These co- or augmenting factors are allergen-independent modulators of the allergic reaction. Particularly extrinsic cofactors such as drugs, alcohol, early phases of infectious diseases and clinically mild infections exert non-immunological modulating effects on type-I reactions (14). Extreme heat and humidity may function in a similar way.

### Diagnosing EIA

EIA and FDEIA are clinically-based diagnoses that depend on taking meticulous anamnesis to identify the events that precipitate the reaction and the involved cofactors, performing provocation testing, and excluding other mimicking conditions. Cholinergic urticaria, primary food allergy exacerbated by exercise, cold-induced urticaria/anaphylaxis, mastocytosis and mast cell activation syndrome, cardiovascular events, exercise-induced bronchoconstriction, and postural orthostatic tachycardia syndrome should be considered and excluded (17). Cholinergic urticaria was excluded clinically as urticarial rash in this patient were pruritic hives (wheals) that are large in size, not pinpoints, with no surrounding large erythema, and it never came on taking a hot bath, as in the cholinergic urticaria (18). Partial immersion in hot water (42°C) test to exclude cholinergic urticaria needs to discontinue antihistamine tablet for 5–7 days before the test, and this cannot be done as the patient was very anxious because of the frequent episodes of anaphylaxis.

If EIA is suspected, it must be determined if it is food-dependent (FDEIA) or not, as this is crucial to the prevention of further attacks (17). Our patient was assessed, with negative outcomes, for mimicking conditions and the impact of eating before exercise. In our patient, we did not perform an exercise challenge test. When the patient first presented at our center, the history unequivocally pointed to EIA and excluded mimicking conditions; plus, her attacks were very infrequent, and she responded well to avoidance of strenuous exercise. Later, when her attacks became more frequent and severe, the patient was not keen to do an exercise challenge test. However, she agreed to an exercise test, done by her cardiologist to exclude ischemic changes and did not develop anaphylaxis symptoms during this test. This negative result was somewhat expected as the test performed did not aim to confirm EIA, and has low sensitivity to do so and was done in a controlled temperature and humidity room, whereas anaphylaxis in real life for this patient occurred in a humid and hot environment.

### Management and prognosis of EIA

The management of EIA includes the identification of relevant cofactors such as NSAIDs and alcoholic beverages and avoiding exposure before exercising, skipping exercising when it is too hot and humid or too cold, avoiding exercising indoors if pollen and/or mold counts are high, always carrying a cell phone, staying close to emergency facilities, wearing medical alert identification, always keeping an epinephrine autoinjector readily available, exercising with a partner who is familiar with recognizing anaphylaxis and with the use of an epinephrine autoinjector, and stopping exercise at the early onset of symptoms (17, 18). Our patient applied all these measures but was unable to achieve good control.

Prophylactic use of oral non-sedating anti-H1-antihistamines is common practice, although there is little supporting scientific evidence. Clinical experience suggests that antihistamines may reduce the severity of episodes of anaphylaxis in some patients but do not prevent symptoms and may mask early dermatological symptoms and delay recognition of anaphylaxis. In FDEIA, anti-H2 antihistamines may also interfere with the normal digestion of food allergens and enhance their intestinal absorption (17, 18). There are no studies evaluating the use of oral corticosteroids or leukotriene-modifying agents in EIA or FDEIA. Sodium cromolyn has been reported to be effective. Based on limited data, cromolyn, in food-related anaphylaxis including FDEA, is most effective when used 20–30 min before meals (17, 18). When anaphylaxis occurs, adrenaline is the mainstay and first line of treatment in EIA, similar to other types of anaphylaxis (1). The patient and his/her exercise partner should be trained to administer adrenaline autoinjectors correctly.

Omalizumab, a monoclonal antibody against immunoglobulin E (IgE), is approved for the treatment of moderate to severe persistent uncontrolled asthma despite maximum asthma treatment in patients with age of 6 years or older, nasal polyposis not controlled by nasal corticosteroids in patients with age of 18 years or older, and antihistamine-refractory chronic spontaneous urticaria (CSU) in patients with age of 12 years or older (19). The use of omalizumab to treat anaphylaxis of various causes, such as idiopathic, venom-induced anaphylaxis, and linked to systemic mastocytosis, was reported (17). Furthermore, using omalizumab in food allergy was previously successful and improved oral immunotherapy efficacy in multifood allergic patients (20, 21). There are only very few case reports of successful treatment of EIA with omalizumab (10–12). Peterson and Coop described a patient with complete resolution of symptoms, which recurred upon discontinuation of omalizumab. Upon reinitiation of omalizumab treatment, the patient showed complete response and remained symptom-free on omalizumab for more than 5 years (10). The precise mechanisms of action of omalizumab in EIA remain unclear and should be explored in further studies. Our patient received 5 doses of omalizumab 300 mg SC every 4 weeks and has been free of anaphylaxis attacks since the start of this treatment.

**Figure 2:**
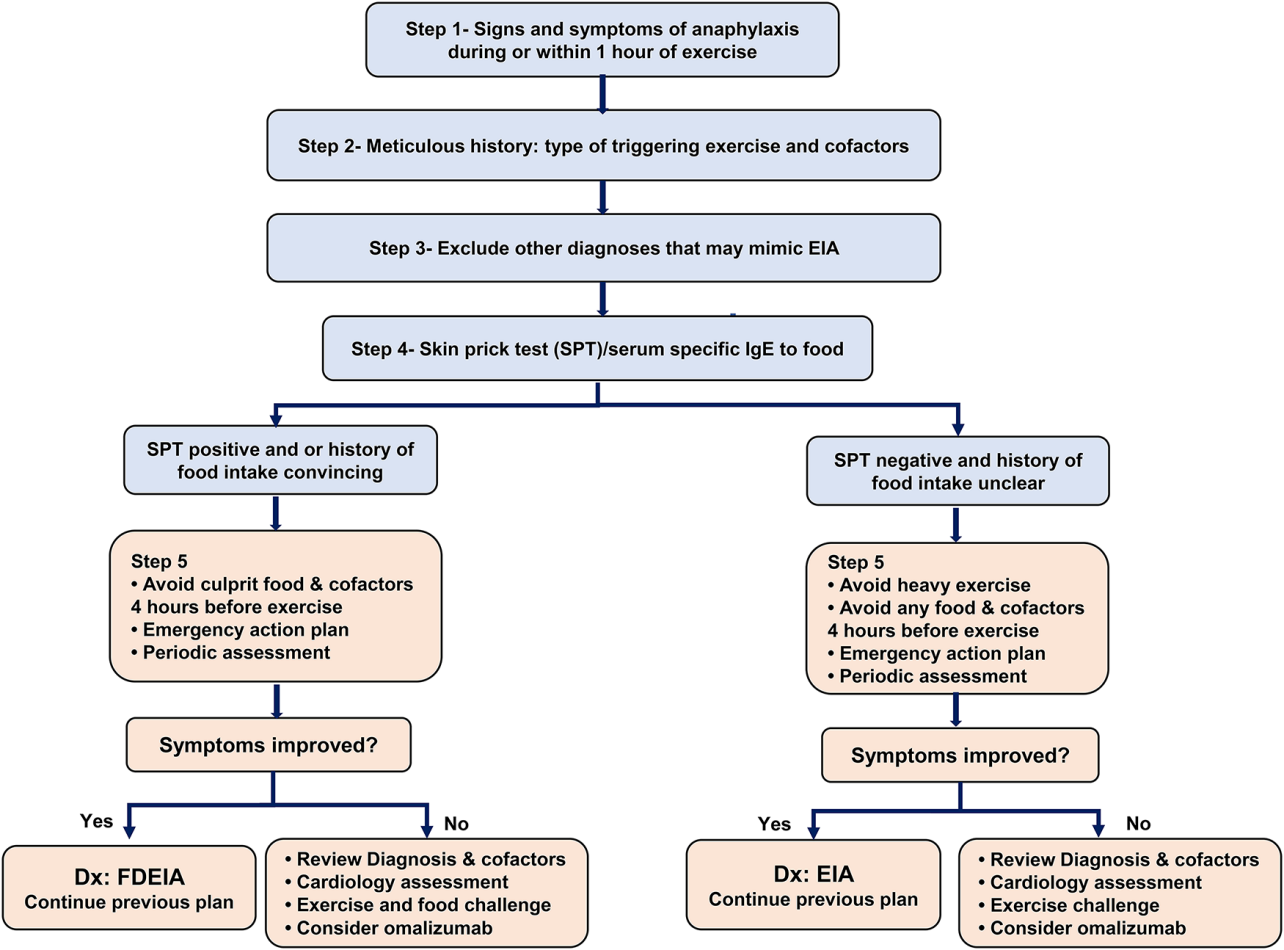
Management of exercise-induced anaphylaxis: step 1- confirming signs and symptoms of anaphylaxis (wheals/angioedema with involvement of one or more of the following 3 systems: respiratory, cardiovascular, and gastrointestinal). Step 2- Meticulous history, documenting the triggering exercise (e.g., running, football, dancing), the intensity of exercise, the time to the onset of anaphylaxis after the start of exercise, cofactors that precede or are present during exercise (e.g., food ingestion, alcohol, NSAIDs intake, extreme weather, and or pollen season). Step 3- Exclude other conditions that mimic EIA (Cholinergic urticaria, primary food allergy exacerbated by exercise, cold-induced urticaria/anaphylaxis, mastocytosis, cardiovascular events, exercise-induced bronchoconstriction, and postural orthostatic tachycardia syndrome) by detailed history, spirometry, baseline serum tryptase and other investigations as indicated. Step 4- Allergy skin prick test (SPT) or specific IgE blood tests for food (culprit food if clear by history, or common food known to trigger FDEIA such as wheat, peanut, tree nuts, fish, shellfish, legumes. Aeroallergens, particularly if they contaminate food (e.g., mold) (22) can be tested as a potential culprit. Step 5- Preventive measures.

Information on EIA is limited, and guidance is needed for its clinical management. Based on published cases, findings from our case, and our experience, we suggest following a stepwise approach in the diagnostic workup and management of patients with signs and symptoms of anaphylaxis after exercising (Figure 2).

As this is a single case study, it has some limitations that must be addressed. There is no provocation testing, which might help implement EIA treatment, and we acknowledged it as a limitation in this report; however, the patient was reluctant to go for that. Additionally, the relatively short follow-up on anti-IgE treatment is another limitation despite an excellent response shown as early as a week after the first dose. Clearly, our case demonstrated that more studies and information on EIA are needed. These studies should further explore the current understanding of the pathophysiology of EIA. The signs and symptoms of EIA are held to result from the activation of mast cells and their release of various mediators, based on skin biopsies showing mast cell degranulation (18) and demonstration of transient elevations in serum tryptase after episodes (23). However, the mechanisms of exercise-induced mast cell activation in EIA are not well understood, and future studies are needed for their identification and characterization. The natural history of EIA shows that about half of the patients improved within a 10-year follow-up: symptoms decreased, became stable, and remained inactive in the last year of observation in 47%, 46%, and 41% of patients, respectively (16). However, the mechanisms of spontaneous remission of EIA and its predictors are unknown and should be addressed by future research.

## Conclusion

EIA is a rare disorder presenting with signs and symptoms of anaphylaxis during or shortly after exercise. The exact pathogenesis is not yet fully understood. It is a clinical diagnosis of exclusion obtained by a meticulous history. Preventive measures include the avoidance of identified triggers and cofactors, exercising with a partner, as well as early treatment of the reaction using an epinephrine autoinjector. In severe recurrent cases, omalizumab can be very helpful, as it was in our patient reported here.

## Data Availability

The original contributions presented in the study are included in the article, further inquiries can be directed to the corresponding author.
